# From digital escape to memory impairment: The mediating role of digital dementia in the effect of digital fugue on digital amnesia

**DOI:** 10.1111/papt.70077

**Published:** 2026-05-15

**Authors:** Deniz S. Yorulmaz‐Demir, Yalçın Kanbay

**Affiliations:** ^1^ Nursing Department, Faculty of Health Sciences Artvin Çoruh University Artvin Turkey; ^2^ Department of Psychiatric Nursing, Faculty of Health Sciences Artvin Çoruh University Artvin Turkey

**Keywords:** amnesia, dementia, digital technology, fugue, mediation analyses, mental health

## Abstract

**Aim:**

This study aimed to analyse the mediating role of digital dementia in the relationship between digital fugue and digital amnesia.

**Method:**

The study sample consisted of students aged 18 and older who were enrolled at a state university in Turkey (*n* = 394). Data were collected through face‐to‐face interviews using the following forms: ‘Personal Information Form’, ‘OPIZA Digital Fugue Scale’, ‘Digital Dementia Scale—Adult Form’ and ‘Digital Amnesia Scale—Adult Form’.

**Results:**

The mean age of the participants was 21.17 ± 1.72 (min: 18, max: 30), 59.4% were female, and the daily internet usage time was 6.24 ± 2.43 h (min: 1, max: 18). The study findings revealed that digital fugue was a significant predictor of both digital dementia and digital amnesia, and digital dementia had a positive and substantial effect on digital amnesia (*p* < .001). The findings also indicated a significant total effect of digital fugue on digital amnesia; however, the inclusion of digital dementia in the model reduced the direct effect but remained significant. According to the bootstrap analysis results, the study identified the indirect effect as statistically significant, and the confidence interval did not contain a ‘zero’ value.

**Conclusion:**

The study concluded that digital dementia partly mediates the relationship between digital fugue and digital amnesia. These results indicate that digital escapism behaviours should be considered in conjunction with cognitive functions and memory processes.

## INTRODUCTION

The unprecedented pace of digital technologies in the 21st century has led to substantial changes and transformations at both individual and societal levels. The expanding availability of internet access, the increased use of smartphones and digital tools, and the advancing quantity and use of social media platforms are among the most significant developments observed in this century. Such advancements in digital technologies have made access to information easier and have improved the quality of life (Farkaš, 2024). However, these opportunities offered by digital technologies and the internet have also generated new and complex risk areas. Numerous studies revealed that the intensive, excessive and misuse of these technologies resulted in several issues, including weakening in social interaction, a decline in academic performance, sleep problems, musculoskeletal problems, perceptions of loneliness, experiences of cyberbullying and behavioural addiction patterns (Dhale et al., 2024; Lissak, 2018; Montag et al., 2021). Beyond these consequences arising from digital technologies, several recently published studies have sparked debates over the severe and adverse effects of digital technologies on cognitive and neurobiological systems (Farkaš, 2024). Numerous studies also emphasize that digital technologies and the internet can lead to lower executive functions, negative impacts on memory processes, increased cognitive load, attention deficits and concentration problems (Clemente‐Suárez et al., 2024; Shafaqat & Sharif, 2023). These findings indicate that the internet and digital technologies are not merely a means of communication but also environmental determinants with significant impacts on neurobiological systems, mental health and psychosocial well‐being. As a result, understanding the impacts of digital technologies and identifying their potential risks early on will be essential in promoting healthy usage patterns and protecting individual and public health.

Digital fugue, digital dementia and digital amnesia have been three key concepts that stand out in the literature, coinciding with the frequent discussions about the effects of digital technologies on neurobiological systems, mental health and psychosocial health. While the concept of digital fugue is defined as ‘individuals consciously distance themselves from their physical or social environment by turning to the digital world and escaping reality through digital tools’, it encompasses not only social withdrawal but also the evolution of cognitive processes and attention to digital stimuli (Kanbay, Akkurt Yalçıntürk, et al., 2025). Digital fugue impairs individuals' psychosocial functioning and lays the groundwork for issues, including distractibility, social isolation and digital addiction (Elhai et al., 2020; Montag et al., 2021). As initially introduced to the literature by German neurologist Manfred Spitzer (2012), the concept of digital dementia was defined as ‘experiencing a decline in attention, memory, learning and thinking skills in individuals due to excessive use of digital tools’ (Spitzer, 2012). Various subject‐related studies suggest that constant stimulation generated by digital environments leads to brain passivity and a decrease in cognitive reserve (Loh & Kanai, 2016; Sigman, 2017). This condition, particularly manifest in young individuals, is considered a cause for concern for researchers due to its structural resemblance to classic dementia symptoms (Kuss et al., 2018). The concept of digital amnesia, however, refers to a context in which individuals tend to store information rather than recall it due to easier access; hence, this context leads to weak long‐term memory (Kaspersky Lab, 2015). This concept, also referred to as the ‘Google effect’, is associated with a decline in individuals' tendency to use their mental memory (Sparrow et al., 2011). Various studies reported that excessive interaction and use of digital settings lead to weakening of individuals' short‐term memory performance, which adversely affects academic achievement (Storm et al., 2017; van Endert, 2021). When these three key concepts, which can be viewed as reflections of the digital world, are assessed collectively, it is noteworthy that the relationship individuals establish with digital technologies manifests profound marks not only on addiction but also on areas such as mental health, attention, memory and social participation. As a result, studying the implications of these three fundamental concepts can provide a novel perspective for a comprehensive interpretation of various psychological issues beyond technology addiction.

### Background

#### The relationship between digital fugue and digital dementia

With the increase in internet access and the integration of digital technologies into daily life, it is well recognized that while the trend of face‐to‐face communication is declining, communication processes are accelerating, especially as a sizable share of social interaction shifts to online platforms (Barros, 2024). Beyond changes in communication processes, it is also known that individuals have delegated their daily life activities to digital technologies or perform activities through digital technologies (Baron, 2021). While changes in digital transformation and communication structures improve quality of life, these practices potentially evolve into escapist usage patterns in time and lead to the evolution of cognitive processes towards digital stimuli (Baron, 2021; Barros, 2024; Farkaš, 2024). When individuals turn to smartphones, social media or digital games, especially during times of anxiety, stress and social pressure, and become detached from their environment, these situations weaken their psychosocial functioning and thus lay the groundwork for issues related to attention deficit, social isolation and digital addiction (Elhai et al., 2020; Montag et al., 2021).

During a digital fugue, individuals are exposed to various digital stimuli, struggling with concentration difficulties and maintaining attention on a single cognitive process (Firth et al., 2019). The hyper‐connectivity and interactions offered by digital settings may disrupt mental continuity, increase cognitive load and exceed the working capacity of memory, leading to underperformance, lower cognitive efficiency and cognitive decline. Over time, cognitive decline may lay the groundwork for the manifestation of digital dementia symptoms, characterized by conditions including mental disarray, difficulty recalling information and amnesia (Baron, 2021; Spitzer, 2012). In other words, an over‐reliance on the digital environment may impair cognitive function, leading to the emergence of dementia‐like symptoms. The subject‐related literature reported that high levels of digital socialization are positively correlated with avoidant coping strategies (Mota et al., 2021), that the internet is a typically used method for coping with negative situations, that dysfunctional negative emotions are associated with extensive internet use and that maladaptive coping strategies increase as internet use increases (Costescu et al., 2021). Another systematic study analysing distractions in educational processes reported that digital tools were among the leading distractors, and performance issues and ineffective classroom teaching are the main outcomes of this context (Martin et al., 2025). These theoretical explanations and research findings in the literature suggest that digital fugue may be a significant predictor of digital dementia through the resulting impairments in cognitive functions. Consequently, the present study hypothesized the following: ‘H1: Digital fugue significantly predicts digital dementia’.

#### The relationship between digital dementia and digital amnesia

Extensive use of digital technologies can lead to cognitive decline, such as difficulty concentrating, recall problems and weakened memory functions, potentially paving the way for the emergence of symptoms of digital dementia. Digital dementia affects encoding, storage and retrieval processes by reducing individuals' capacity to actively retain and recall information (Spitzer, 2012). These cognitive shifts may lead individuals to view information as something that can be re‐accessed through digital technologies rather than something recalled mentally; hence, it may lead to an increased tendency to rely on digital‐based recall, causing difficulties in accessing information without digital devices (Kaspersky Lab, 2015). To put it differently, the impairments in memory performance associated with digital dementia may lead individuals to delegate the responsibility for the recalling process to digital devices, potentially resulting in the development of digital amnesia over time.

The relevant literature reports that the excessive interaction and addiction to digital settings weaken individuals' short‐term memory performance and negatively impact academic achievement (Storm et al., 2017; van Endert, 2021); that individuals who believe they can re‐access information through digital technologies are less likely to recall it (Sparrow et al., 2011) and that internet use increases the tendency towards superficial information processing (Marsh & Rajaram, 2019). Indeed, a study on Finnish adults found that the perception of memory loss was approximately twice as common among adults as their duration of internet usage increased (Näsi & Koivusilta, 2013). Another experimental study, in which individuals of various age groups were observed, reported that the speed of text memorization decelerated as the duration of internet usage increased (Cheremoshkina, 2011). A qualitative study analysing experiences with smartphone applications also documented that individuals created notes and voice alerts on their smartphones to recall several activities during the day (García et al., 2016). In this sense, it is reasonable to consider such relationships between digital dementia and digital amnesia within the context of how the decline and weakening of cognitive functions are reflected in memory processes. As a result, addressing the ramifications of digital technologies within a theoretical framework may offer substantial insights into protecting oneself from the consequences of the digital world. In line with conclusions and justifications in the literature, the present study hypothesized the following: ‘H2: Digital dementia significantly predicts digital amnesia’.

#### The relationship between digital fugue and digital amnesia

Nowadays, individuals use digital technologies for a variety of daily activities, including information searching, learning new things, shopping, visiting museums, studying and reading books, as a result of communication methods evolving into digital environments and technological advancements becoming an essential part of life (Baron, 2021; Barros, 2024). While the use of digital technologies is initially associated with detachment from the environment, withdrawal and social isolation, it may also harm cognitive processes in the long term. During the digital fugue process, individuals use their mental processing and recall skills less frequently due to the conveniences offered by digital technologies, such as constant accessibility and rapid access to information; consequently, they eventually require these abilities less often over time. As a result of this habit, the efficient utilization of memory and cognitive functions may be limited, and mental access capacity may decrease. This cognitive shift in digital fugue prepares the ground for individuals to transfer their memory and recall requirements from internal memory systems to external digital systems, leading them to develop new strategies, such as how and where to access information, rather than simply recalling it.

The ‘we can always call it again’ and ‘the internet will recall it for us’ skills offered by digital technologies can create obstacles in remembering, retrieving and accessing information in settings without these technologies. Indeed, various topic‐related studies report that individuals who believe they can re‐find information through digital technologies are less likely to remember it (Sparrow et al., 2011); individuals tend to be less likely to remember objects they photographed than those they only observed (Soares & Storm, 2018) and the accuracy of spatial information declines as the use of global positioning/navigating systems increases (Hejtmánek et al., 2018). Another study analysing the effects of reading through print and digital media on text processing, comprehension and metacognitive activities reported that individuals exerted less mental effort when reading through digital media (Delgado & Salmerón, 2022). In light of theoretical information and research findings in the literature, digital fugue may be a significant predictor, playing a critical role in the development of digital amnesia. As a result, the present study hypothesized the following: ‘H3: Digital fugue significantly predicts digital amnesia’.

#### The mediating role of digital dementia in the relationship between digital fugue and digital amnesia

A thorough analysis of the relationship between digital fugue and digital amnesia requires considering and understanding the factors that may influence these two conditions. As individuals increasingly rely on digital settings over time, digital fugue may impair cognitive function and memory, potentially leading to the development of ‘digital dementia’. Digital dementia, characterized by weakened cognitive functions and a diminished capacity to store and recall information, can be exacerbated by individuals' increased tendency to access information through digital technologies, leading to the development of digital amnesia. This process implies that digital fugue may have indirect effects through digital dementia in addition to its direct impact on digital amnesia. While the literature includes numerous studies on the effects of digital technologies on cognitive function processes, covering various aspects such as attention span, learning performance and depth of information processing, it is possible to assert that holistic models explaining the consequences of reliance on digital environments on cognitive mechanisms are limited. Furthermore, studies on digital technologies emphasize that despite increasing research volumes, conceptual advancements in the field are limited, and more concept‐based research is needed to fill these research gaps (Kadhiravan & Robert, 2025). Studying these three key concepts, which can be regarded as reflections of the digital world, together may contribute to understanding the multifaceted effects of digital technologies, encourage conscious usage patterns and adopt strategies that facilitate the long‐term sustainability of cognitive functions. Considering the conceptual explanations in the literature, the limitations of theoretical evaluation in research and the significance of a mechanism‐based understanding of cognitive changes related to digital technologies, the present study hypothesizes the following: ‘H4: Digital dementia plays a mediating role in the relationship between digital fugue and digital amnesia’. The findings obtained from this study are expected to contribute to the literature examining the effects of digital technologies from a mechanism‐based perspective and to fill an important gap in the field.

## METHOD

### Research aim

This study aimed to analyse the mediating role of digital dementia in the effect of digital fugue on digital amnesia. Various studies in the literature reportedly analysed the effect of digital device use on cognitive processes; however, the quantity of studies focusing on the memory‐based outcomes of digital escape behaviours was quite limited. As a result, this study addressed the potential consequences of digital escape behaviours on individuals' cognitive functions and memory processes within the framework of a holistic model.

### Research model

This research is a cross‐sectional quantitative study that aimed to analyse the mediating role of digital dementia in the relationship between digital fugue and digital amnesia. Accordingly, the study favoured a digital mediation model to explain the effects of escape behaviours on individuals' cognitive functions and memory processes through a mechanism‐based approach. In the model, the study considered digital fugue, digital amnesia and digital dementia as the independent, dependent, and mediating variables, respectively.

### Population, sample and participants

The population consisted of students studying at a Turkish state university. According to the official records, 17,796 students were enrolled in the university in the 2025–2026 academic year. To evaluate the sample size adequacy, the G*Power 3.1.9 software program was used for a power analysis. For the two‐tailed hypothesis, a small effect size (*f* ^2^ = .05), a significance level of .05 and 95% test power were used as the basis for the analysis. Considering that indirect effects may be relatively small in mediation models, a small effect size was used in the power analysis (Cohen, 2013; Faul et al., 2009). Accordingly, the minimum sample size required for a multiple regression model with two predictor variables was calculated as 262. As a result, the 394 participants in the current study provided an adequate sample size for analyses with high statistical power.

#### Participant features

The mean age of the participants was 21.17 ± 1.72 (min: 18, max: 30). While 59.4% of the participants were female, the vast majority (42.9%) were third‐year students. The average daily internet usage time of the participants was 6.24 ± 2.43 h (min: 1, max: 18). When access to digital devices was restricted, 25.6% of participants reported feeling uneasy, while 15.2% expressed feeling uneasy occasionally. Analysis of smartphone usage during sleep also revealed that 53.0% of participants turned off their phones, 24.6% left them on at night and 22.3% kept their phones on occasionally.

### Inclusion criteria and data collection

The study used the following as inclusion criteria: being an active student at the relevant university, being 18 years of age or older, and volunteering to participate. Research data were collected face‐to‐face within the university campus between November 2025 and January 2026. Researchers evaluated students in the cafeteria, seating area and waiting room based on inclusion criteria, informed them about the research objectives, and invited them to participate in the study. Subsequently, they provided the data collection tool to students who agreed to participate in the study. The participants completed the data collection tools in 5–6 min.

### Data collection tools

This study collected the data using the Personal Information Form, the OPIZA Digital Fugue Scale, the Digital Dementia Scale—Adult Form and the Digital Amnesia Scale—Adult Form.

#### Personal information form

This form aims to reveal several descriptive features of the participants, including age, gender and grade level. It also includes various questions to assess participants' daily internet usage time and digital device usage habits.

#### OPIZA digital fugue scale

This scale aims to assess adults' tendency to distance themselves from social, environmental and emotional stimuli by turning to digital settings and is a self‐report tool consisting of two factors and 10 items (sample item: ‘I use digital devices in environments where I feel social pressure’). It is a 5‐point Likert scale, and a high total scale score indicates a high level of digital fugue. The scale explained 67.6% of the total variance, and Cronbach's *α* and McDonald's Omega values were .81 and .93, respectively (Kanbay, Akkurt Yalçıntürk, Babaoğlu, Akçam, & Tektaş, 2025).

#### Digital dementia scale—adult form

This scale measures cognitive, emotional and daily functioning impairments in adults resulting from digital device usage and prolonged screen time and is a self‐report tool consisting of three factors and 15 items (sample item: ‘I have difficulty concentrating depending on digital device usage’). It is a 5‐point Likert scale, and a higher total score indicates a higher level of digital dementia. The scale reportedly explained 65.7% of the total variance, and the Cronbach's *α* value was .92 (Kanbay, Akkurt Yalçıntürk, Babaoğlu, & Akçam, 2025).

#### Digital amnesia scale—adult form

This scale assesses memory‐based impairments in adults that arise from digital device usage in the processes of accessing, storing and recalling information. It is also a self‐report tool consisting of two factors and 12 items (sample item: ‘I have difficulty recalling information without my digital devices’). It is a 5‐point Likert scale, and a higher total score means a higher level of digital amnesia. The Cronbach's *α* value of the scale was .93 (Kanbay, Babaoğlu, Akkurt Yalçıntürk, & Akçam, 2025).

### Data analysis

IBM SPSS (Statistical Package for the Social Sciences) v.26.0 and IBM AMOS (Analysis of Moment Structures) v.23.0 software packages were used for the data analysis procedure. Researchers checked for missing/outlier data before the analyses, and accordingly, excluded 24 survey datasets with improper responses (missing responses and blank responses). The assumptions of normal distribution, skewness and kurtosis values were also tested. As a result, researchers decided to perform parametric tests since these values fell within the ±1 range.

#### Construct validity

Before initiating the research, Confirmatory Factor Analysis (CFA) was performed to evaluate the construct validity of the measurement tools. CFA is a structural equation model (SEM)‐based analysis technique that allows testing how well the collected data validate the existing construct (scale and sub‐dimensions), and common fit indices, including *χ* ^2^/*df*, RMSEA, CFI, GFI, IFI and SRMR values, are used in the interpretation of CFA analysis (Kline, 2016). Numerous studies in the literature considered the following as critical values for model fit indices: below 5 for *χ* ^2^/*df*, 0.08 and below for RMSEA, 0.90 and above for CFI, GFI and IFI, and 0.08 and below for SRMR (Hair Jr et al., 2019; Kline, 2016). As a result, the present study tested the measurement tools within the framework of these values proposed in the literature.

#### Composite reliability and convergent validity

In addition to the CFA results, the study analysed the Average Variance Extracted (AVE) values to evaluate the composite reliability (CR) and convergent validity of the measurement models. While 0.70 and above CR values indicate sufficient internal consistency of the relevant construct, 0.50 and above AVE values refer to an indication that the scale items adequately represent the relevant latent variable (Fornell & Larcker, 1981; Hair Jr et al., 2019). Considering these values reported in the literature, the present study evaluated the convergent validity and composite reliability of the measurement tools.

#### Common method bias

Since the study data were collected through self‐reporting, the risk of common method bias was assessed. For this purpose, Harman's single‐factor test was initially performed (Harman's single‐factor test was conducted using a free trial method), and the analysis revealed that a single factor explained 32.1% of the total variance. Studies in the literature report that if a single factor fails to explain a substantial portion of the total variance. It is also considered an indication that common method bias does not pose a serious threat to research findings (Podsakoff et al., 2003). The study also performed the full collinearity test proposed by Kock (2015) to analyse common method bias more comprehensively. Accordingly, the analysis revealed that the variance inflation factor (VIF) values of the variables ranged from 1.336 to 1.413. The maximum VIF value was also well below the recommended threshold of 3.3 (Kock, 2015). As a result, the study concluded that common method bias was at a low level (minimal), and there was a low probability that it would significantly skew model estimates.

#### Mediation model

As developed by Hayes (2018), the PROCESS Macro plugin was used to analyse the direct and indirect relationships among variables, and mediation analysis was performed using Model 4 (Hayes, 2018). The bias‐corrected bootstrap method was also used to test indirect effects. In the model, digital fugue, digital amnesia and digital dementia were regarded as the independent, dependent and mediating variables, respectively. The Bootstrap method was used to test the significance of the mediating effect. In this context, 5000 resamples were employed, and the significance of the indirect effect was evaluated at a 95% confidence interval. The absence of a ‘0’ (zero) in the confidence interval indicated a statistical significance for the mediating effect. Lastly, the significance level for all analyses in the study was set at *p* < .05.

### Ethical considerations

Before initiating the study, ethical committee approval (Number: E‐18457941‐050.99‐202044, Date: November 20, 2025) and institutional permission (Number: E‐82587833‐605‐202719, Date: November 26, 2025) were granted by the relevant university where the research was conducted. During the data collection procedure, researchers informed participants about the purpose and content of the research, the principles of voluntariness and their right to withdraw at their convenience. No personal information was requested from the participants during the research process, and the collected data were analysed solely for scientific purposes. This study adhered to the principles in the Declaration of Helsinki during the research process (World Medical Association, 2013). No artificial intelligence tools or automated systems were used in the conceptualization, analysis or verification of this manuscript. AI‐assisted tools (e.g., Grammarly) were used solely for language and grammar editing. The authors take full responsibility for the accuracy, integrity and originality of the content of this manuscript.

## RESULTS

This section presents the statistical analysis results regarding the variables of digital fugue, digital dementia and digital amnesia, based on the data collected during the research process. Accordingly, the section initially reports descriptive statistics, reliability coefficients and correlation values for the variables (using the SPSS program). Subsequently, it presents the CFA results used to evaluate the structural validity of the measurement tools (using the AMOS program). Finally, the section provides the findings of the mediation analysis, which tests the mediating role of digital dementia in the relationship between digital fugue and digital amnesia (using the SPSS program).

Skewness and kurtosis analysis revealed that these values for all variables were within the recommended ±1 limits; hence, the data met the assumption of normal distribution (Hair Jr et al., 2019; Kline, 2016). The Cronbach's *α* reliability coefficient of the measurement tools was also higher than .70 (.89, .93 and .90 for OPİZA Digital Fugue, Digital Dementia and Digital Amnesia, respectively), and the measurements were deemed reliable (Hair Jr et al., 2019; Taber, 2018). Before regression‐based analyses, the assumption of multicollinearity was also tested; accordingly, the VIF (1.352) and tolerance (0.740) values of the independent variable remained within the permissible limits. This data indicates that there is no multicollinearity problem among the variables (Hair Jr et al., 2019). Furthermore, the Durbin–Watson coefficient was calculated to be 2.024, which was used to evaluate the independence of the error terms. This value indicated that the autocorrelation assumption was not violated since the calculated value fell within the recommended range of 1.5–2.5 (Field, 2018). Pearson correlation analysis results revealed that digital fugue was positively and moderately correlated with both digital dementia (*r* = .510, *p* < .01) and digital amnesia (*r* = .501, *p* < .01). A positive and moderately significant correlation was also found between digital dementia and digital amnesia (*r* = .549, *p* < .01). These findings explicitly indicate significant relationships between the variables, and the statistical assumptions required for conducting advanced regression‐based analyses are met (Cohen, 2013; Schober et al., 2018) (Table 1).

**TABLE 1:** Descriptive statistics and hypothesis tests for variables.

Measurement tools	$\bar{x}$	*SD*	SK	KU	1	2	*α*	VIF	T	DW
1. Digital fugue	54.7	19.7	.023	.076	1		.89	1.352	.740	2.024
2. Digital dementia	46.5	19.7	−.108	.091	510**	1	.93			
3. Digital amnesia	50.9	19.8	−.138	.254	.501**	.549**	.90			

*Note*: Correlation coefficients were calculated using the Pearson method; **p < .01.

Abbreviations: $\bar{x}$, mean; DW, Durbin–Watson coefficient; KU, kurtosis; *SD*, standard deviation; SK, skewness; T, tolerance; VIF, variance inflation factor; *α*, Cronbach's alpha.

Following descriptive evaluations, the study analysed the construct validity, convergent validity and composite reliability of the measurement tools. Accordingly, while the calculated fit indices for the OPIZA Digital Fugue Scale (*χ* ^2^/*df* = 3.09, CFI = .96, GFI = .96, IFI = .96, RMSEA = .073, SRMR = .046) indicated that the model displayed a good fit, the fit indices for the Digital Dementia Scale (*χ* ^2^/*df* = 3.26, CFI = .94, GFI = .92, IFI = .94, RMSEA = .076, SRMR = .048) and Digital Amnesia Scale (*χ* ^2^/*df* = 3.60, CFI = .94, GFI = .93, IFI = .94, RMSEA = .081, SRMR = .038) displayed acceptable fits (Hu & Bentler, 1999; Kline, 2016). Analysis of the AVE and CR values of the measurement instruments also revealed that the measurement instruments satisfied the convergent validity and composite reliability criteria (OPIZA Digital Fugue Scale: AVE = .62, CR = .78; Digital Dementia Scale: AVE = .64, CR = .81; Digital Amnesia Scale: AVE = .54, CR = .74) (Fornell & Larcker, 1981) (Table 2). As a result, these findings led to the conclusion that all the study's measurement tools provided valid and reliable measurements on the current sample.

**TABLE 2:** Confirmatory factor analysis results for measurement tools.

Measurement tools	*χ* ^2^/*df*	CFI	GFI	IFI	RMSEA	SRMR	AVE	CR
Digital fugue	95.834/31	0.96	0.96	0.96	0.073 [0.057/0.090]	0.046	0.62	0.78
Digital dementia	280.315/86	0.94	0.92	0.94	0.076 [0.066/0.086]	0.048	0.64	0.81
Digital amnesia	176.619/49	0.94	0.93	0.94	0.081 [0.069/0.095]	0.038	0.54	0.74

*Note*: The AMOS software package was used for CFA analyses. AVE and CR values were calculated based on the final measurement models.

Abbreviations: *χ* ^2^/*df*, chi‐square/degrees of freedom; AVE, average variance extracted; CFA, confirmatory factor analysis; CFI, comparative fit index; CR, compound reliability; GFI, goodness‐of‐fit index; IFI, incremental fit index; RMSEA, root mean square errors of approximation; SRMR, standardized mean square errors.

The present study tested the proposed hypotheses within the framework of mediation analysis. Accordingly, Table 3 provides these findings.

**TABLE 3:** Relationships among variables and the mediating role of digital dementia.

Effect	*b*	*SE*	*t*	*p*	[LLCI, ULCI]
X → M	.511	.043	11.750	.000	[0.426; 0.597]
M → Y	.385	.047	8.184	.000	[0.293; 0.478]
Total effect (X → Y)	.503	.044	11.475	.000	[0.417; 0.589]
Direct effect (X → Y)	.306	.047	6.489	.000	[0.213; 0.399]
Indirect effect (X → M → Y)	.197	.045	–	–	[0.114; 0.288]

*Note*: The bootstrap method was used to test indirect effects (5000 samples, 95% confidence interval) as proposed by Hayes (2018). The absence of zero in the confidence interval indicates that the indirect effect is statistically significant.

Abbreviations: *b*, non‐standardized regression coefficient; LLCI, lower limit confidence interval; M, digital dementia; *p*, significance level; *SE*, standard error; *t*, *t*‐statistic; ULCI, upper limit confidence interval; X, digital fugue; Y, digital amnesia.

### Results on digital fugue's ability to predict digital dementia (H1)

The first research hypothesis (H1) proposed that digital fugue significantly predicts digital dementia. The regression analysis results revealed that digital fugue had a positive and statistically significant effect on digital dementia (*b* = .511, SH = .043, *t* = 11.750, *p* < .001, 95% CI = [0.426, 0.597]). Considering the non‐standardized regression coefficient, a one‐unit increase in the digital fugue score corresponds to approximately a .51‐unit increase in the digital dementia score. This data demonstrates that as the tendency to distance from environmental and social stimuli by turning to digital settings increases, cognitive, attention and functional impairments related to digital device use have significantly increased among individuals. The absence of a zero value in the effect's confidence range also indicates that the nature of this relationship is stable and independent of sampling error (Hayes, 2018). According to these results, *the H1 hypothesis was accepted*.

### Results on digital dementia's ability to predict digital amnesia (H2)

The second research hypothesis (H2) proposed that digital dementia significantly predicts digital amnesia. Indeed, analysis results revealed that digital dementia predicted digital amnesia positively and significantly (*b* = .385, SH = .047, *t* = 8.184, *p* < .001, 95% CI = [0.293, 0.478]). The calculated regression coefficient also associated a one‐unit increase in digital dementia level with an approximately 0.39‐unit increase in digital amnesia score. Hence, this data indicates that distraction (attention deficit), cognitive fatigue and mental disarray resulting from digital device use lead to significant impairment in individuals' ability to recall information and the memory processes. A narrow and far from zero confidence interval also supports the strong and consistent nature of this relationship. As a result, *the H2 hypothesis was accepted*.

### Results of the total effect of digital fugue on digital amnesia (H3)

The third hypothesis (H3) assumed that digital fugue significantly predicts digital amnesia. Accordingly, the findings revealed that the total effect of digital fugue on digital amnesia is statistically significant (*b* = .503, *SE* = .044, *t* = 11.475, *p* < .001, 95% CI = [0.417, 0.589]). According to this coefficient, a one‐unit increase in the level of digital fugue corresponds to approximately a 0.50‐unit increase in the digital amnesia level. This finding revealed that digital escape behaviours affect individuals' memory processes both directly and indirectly. As the tendency to use digital settings increases, it is reasonable to assert that individuals' abilities to recall information, retain mentally and actively use memory processes decrease. As a result, *the H3 hypothesis was accepted*.

### Results about the mediating role of digital dementia (H4)

The fourth research hypothesis (H4) predicted that digital dementia plays a mediating role in the relationship between digital fugue and digital amnesia. Accordingly, the mediation analysis, performed to test this hypothesis, confirmed that digital dementia is a statistically significant mediator in this relationship. The inclusion of the mediating variable in the model reduced the direct effect of digital fugue on digital amnesia; however, the effect was still significant (*b* = .306, SH = .047, *t* = 6.489, *p* < .001, 95% CI = [0.213, 0.399]). This data indicates that digital dementia is a partial mediator (Zhao et al., 2010). The indirect effect test using the bootstrap method also revealed that the indirect effect of digital fugue on digital amnesia was significant (*b* = .197, SH = .045, 95% CI = [0.114, 0.288]). The absence of zero value in the confidence interval indicates that the indirect effect is statistically significant (Hayes, 2018; Preacher & Hayes, 2008). According to these results, a one‐unit increase in digital fugue level results in an additional increase of approximately 0.20 units in digital amnesia level via digital dementia. In other words, digital escape behaviours affect individuals' memory processes both directly and indirectly through impairments observed in cognitive functions. The sustained significance of the direct effect suggests that the relationship between digital fugue and digital amnesia cannot be explained by a single mechanism, indicating that cognitive and behavioural processes operate simultaneously. As a result, *the hypothesis H4 is accepted*.

**FIGURE 1:**
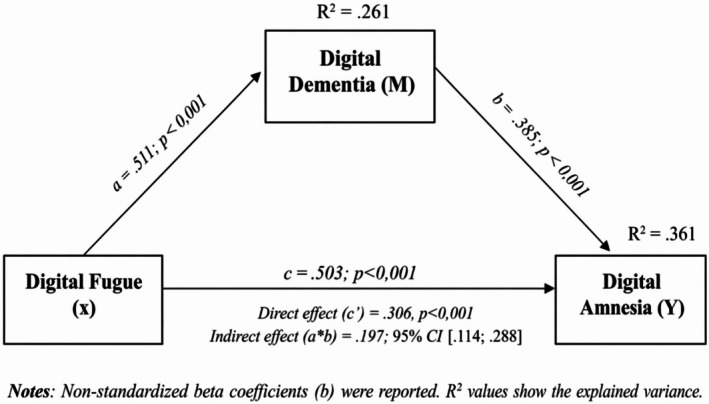
Mediating role of digital dementia (Hayes, Model 4).

Figure 1 displays the path coefficients for the mediation model. Analysis of the standardized path coefficients for the mediation model reveals that digital fugue has a strong and positive effect on digital dementia (*β* = .51). The effect of digital dementia on digital amnesia was also significant and positive (*β* = .38). The inclusion of the mediating variable in the model reduced the direct effect of digital fugue on digital amnesia but retained its significance (*β* = .31). Furthermore, analysis of the variance explained by the model revealed that while digital fugue explained 26% of the variation in digital dementia, the variables included in the model explained 36% of the variance in digital amnesia. These findings explicitly supported the idea that digital dementia partially mediates this relationship (Figure 1).

## DISCUSSION

This study revealed the mediating role of digital dementia in the effect of digital fugue on digital amnesia, analysing the cognitive consequences of digital behaviours within the framework of a mechanism‐based model. The study findings indicated that digital fugue was a substantial predictor of digital dementia, and digital dementia was a strong determinant of digital amnesia. Additionally, the significant indirect effect suggested that digital escape behaviours might affect memory processes not directly, but rather through weakened cognitive functions. These results indicated that digital behaviours should be analysed through cognitive mediators in addition to habit or frequency of use.

### The effect of digital fugue on digital dementia

One of the key conclusions of this study is that digital fugue is a strong predictor of digital dementia, which represents impairments in cognitive function. This relationship can be explained within the framework of the theory of limited cognitive resources. The human cognitive system has a limited attention capacity, and constant exposure to stimuli potentially leads to the gradual depletion of these limited resources (Kahneman, 1973). Indeed, the relevant literature reports that even if individuals inactively use their smartphones, their mere physical presence may hinder cognitive capacity, resulting in a decline in performance on attention‐requiring tasks (Skowronek et al., 2023; Ward et al., 2017). These findings provide a significant theoretical basis for understanding the cognitive process experienced by individuals who turn to digital fugue. In the context of digital fugue, individuals are constantly compelled to regulate their attention between digital and environmental stimuli, rather than completely detaching from them. Although this context may appear to be an escape behaviour on the surface, it actually causes the cognitive system to experience a chronic and invisible cognitive burden. Constantly suppressed or delayed attention focusing may reduce the effectiveness of executive functions and, in the long term, exacerbate the symptoms of digital dementia, characterized by a decline in cognitive efficiency. From this perspective, it is reasonable to consider digital fugue not only a behavioural avoidance pattern but also a risk mechanism in which cognitive resources are continuously depleted.

Despite some studies in the relevant literature indicating that smartphones may reduce cognitive capacity and cause a decrease in performance on attention‐demanding tasks, even when not actively used (Skowronek et al., 2023; Ward et al., 2017), some ecologically valid studies suggest that the effects of smartphones on cognitive performance may be more limited and context‐sensitive (Hartanto et al., 2024). For instance, in two separate experimental studies conducted in natural classroom settings, le Roux et al. (2025) were unable to report concrete and consistent evidence that the presence of a smartphone on a student's desk significantly reduced fluid‐thinking performance (le Roux et al., 2025).

Such varying results suggest that cognitive consequences attributed to the ‘mere presence’ of a smartphone should be viewed not as universal and direct effects, but as interactions with individual orientations and cognitive regulatory processes. At this point, digital fugue offers an explanatory framework for interpreting these contradictory results in the literature. Individuals who turn to the digital fugue should actively regulate their attention between digital and environmental stimuli rather than completely detaching themselves from them. Hence, it may not lead to a significant decline in performance in the short term, especially in structured classroom environments with high levels of external control. However, this constant need for attention regulation potentially leads to a chronic cognitive load in the long term, exacerbating symptoms of digital dementia, characterized by weakened executive functions and decreased cognitive efficiency. In this context, the findings of le Roux et al. (2025) indicate that real‐time performance measurements based solely on smartphone location may not fully reflect cognitive risks, suggesting that recurring patterns of attention and avoidance, such as digital fugue, may play a more significant role in understanding cognitive decline (le Roux et al., 2025). Consequently, digital fugue should be considered not merely as a physical manifestation of the smartphone but as a risk mechanism that operates through the way an individual regulates their attention and has long‐term cognitive consequences.

### The effect of digital dementia on digital amnesia

Another key conclusion of the study is that digital dementia is a powerful predictor of digital amnesia. This result also aligns with theoretical perspectives that propose digital technologies reorganize cognitive processes, and especially with the literature on cognitive offloading (Ali et al., 2024; Grinschgl et al., 2021). Excessive digital media use and extended screen time negatively impact brain development, particularly in areas essential for adapting to dynamic environments, including cognitive and inhibitory control, attention, memory and reasoning. Early exposure to fast‐paced social media may impair motor skills, spatial awareness, problem‐solving skills and language acquisition. Studies reveal that excessive smartphone addiction is associated with a decrease in grey matter in some key brain regions, potentially impairing cognitive and emotional regulation (Ali et al., 2024; Wallace et al., 2023). The widespread use of the internet and digital tools in daily life has been shifting the way individuals mentally encode and retain information and prioritizing metacognitive strategies for finding it. Indeed, as proposed by Sparrow et al. (2011), the ‘Google effect’ suggests that individuals tend to remember the methods of accessing information rather than the content itself when they are aware that it is readily accessible (Sparrow et al., 2011).

Digital dementia can be considered not only a general weakening of cognitive functions but also a process characterized by the systematic transfer of memory functions to digital environments. Experimental data by Grinschgl et al. (2021) revealed that cognitive externalization potentially improves task performance in the short term; however, it degrades memory formation in the long term (Grinschgl et al., 2021). Hence, the cognitive mechanism learns to ‘entrust’ information to technological instruments rather than organizing it internally, which explains why individuals with a higher level of digital dementia are more prone to display digital amnesia symptoms. This process gains even more significance in the context of transactive memory theory. As Wegner (1995) proposed, the transactive memory model divides the memory load of individuals among social or technological systems (Wegner, 1995). Current studies also report that Internet use reorganizes this system at the expense of individual memory; hence, individuals consider the Internet a primary source for their future information demands (Storm et al., 2017). Such reorganization diminishes individuals' confidence in their memory while increasing their reliance and dependence on digital systems. As a result, this context theoretically supports the predictive role of digital dementia on digital amnesia.

Yet, holistic approaches to how the internet affects cognitive processes also support these findings. Recent psychological and neuroscientific studies have reported that the internet might lead to both temporary and permanent changes in attention, memory and social cognition (Firth et al., 2019, 2020; Korte, 2020; Manwell et al., 2022). The continuously updated online information flow weakens sustained attention; additionally, the constant accessibility of information transforms the storage and retrieval functions of memory. Such a transformation could have critical consequences for cognitive development, especially among the youth, indicating that the relationship between digital dementia and digital amnesia is not only an individual dimension but also a developmental and societal one.

In conclusion, the present study findings indicate that the prediction of digital amnesia by digital dementia should be considered not solely on the basis of attention or amnesia but as a combined effect of cognitive externalization, transactive memory reorganization and internet‐based cognitive transformation processes. As digital dementia increases, individuals' motivation to actively use their memory decreases; as a result, digital amnesia easily develops and becomes permanent. Therefore, it is reasonable to assert that digital amnesia is both a consequence of digital dementia and an inevitable reflection of digitized cognitive architecture.

### The effect of digital fugue on digital amnesia

Another significant finding of the study was that digital fugue had a substantially positive total effect on digital amnesia. This finding reveals that individuals' tendency to use digital settings to escape social, environmental or emotional stimuli is associated with significantly weakened memory‐based functions. A one‐unit increase in digital fugue levels corresponds to approximately a half‐unit rise in digital amnesia levels, suggesting that this relationship is significant not only statistically but also behaviourally and cognitively. Individuals who turn to digital fugue may develop a tendency to search for and access information in digital settings rather than mentally retaining, internalizing and recalling it in their daily lives. This context also coincides with the process described in the literature as ‘cognitive offloading’ (Risko & Gilbert, 2016). The Google effect, reported by Sparrow et al. (2011), specifically indicates that when individuals have constant access to information, they tend to remember where to find it rather than recalling the data itself (Sparrow et al., 2011). This phenomenon becomes even more pronounced in the context of digital fugue, as individuals transfer not only access to information but also the very use of memory itself to the digital setting. Therefore, digital fugue can be considered not merely a passive state of distraction but rather a form of conscious or semi‐conscious withdrawal from memory processes.

The direct effect of digital fugue on digital amnesia demonstrates that cognitive capacity, as well as behavioural preferences and coping mechanisms, form the memory processes. While digital escape behaviour often appears to be a functional strategy that reduces immediate stress or protects against social pressure, it may lead to cognitive inertia by lowering the active use of memory processes in the long term. This finding is consistent with studies reporting that avoidance‐based coping strategies, despite their short‐term gains, may have long‐term cognitive consequences (Holahan et al., 2005). This finding also suggests that digital amnesia should be considered not simply as ‘forgetfulness’ or ‘memory weakness’ but also as a behavioural tendency based on avoiding the use of memory. Digital fugue serves as a foundation for individuals to systematically delegate responsibility for their memory processes to digital settings, which may eventually lead to a decrease in the effort to recall and a functional atrophy of memory use over time (Storm et al., 2017).

### The mediating role of digital dementia in the relationship between digital fugue and digital amnesia

As another significant outcome, this study found that digital dementia played a statistically significant mediating role in the relationship between digital fugue and digital amnesia. The significant indirect effect of digital fugue on digital amnesia, and the absence of a zero‐valued confidence interval, suggest that digital escape behaviours influence impairments in memory processes and cognitive functions. The inclusion of the mediating variable in the model reduced the direct effect of digital fugue on digital amnesia but remained significant, indicating that digital dementia partially mediated this relationship (Hayes, 2018; Preacher & Hayes, 2008). According to Zhao et al. (2010), these findings demonstrate that the relationship between variables cannot be explained by a single psychological mechanism and that multiple processes are operating simultaneously (Zhao et al., 2010). As a result, Digital amnesia can be considered not only a direct result of digital escape behaviour but also an outcome of cognitive decline arising from digital device use.

The mediating role of digital dementia highlights the essence of focusing on cognitive mediators in explaining the effect of digital fugue on memory. Individuals experiencing digital fugue turn to digital media to escape stressful or socially demanding environments, and this tendency may impair attention, mental continuity and executive functions over time. Attention and executive functions, on the other hand, play a critical role in encoding information and transferring it to long‐term memory (Baddeley, 2000). Consequently, digital dementia serves as a functional cognitive bridge explaining the relationship between digital fugue and digital amnesia. This mechanism also aligns with the limited cognitive resource approach. When an individual's attention and cognitive control resources are constantly focused on digital stimuli, the resources available for other mental processes decrease, leading to a decline in cognitive performance (Kahneman, 1973; Small et al., 2020). Additionally, the mediation‐related results suggest that digital amnesia is not merely a decrease in an individual's memory capacity, but rather a reflection of a general impairment in cognitive functions manifesting in memory processes. In this sense, digital dementia stands out as a key concept that clarifies memory impairment in the digital era. While digital fugue serves as an escape strategy that shields individuals from environmental constraints in the short term, it erodes the durability of cognitive functions in the long term. However, this weakening generates a fundamental basis that fuels digital amnesia. This finding adds a substantial theoretical contribution to models that clarify the psychological effects of digital behaviour. While most studies directly link digital escape behaviours to outcome variables, the current studies emphasize that these relationships should be addressed in an indirect and process‐based manner. The mediating role of digital dementia reveals that the relationship between digital fugue and digital amnesia is not a linear and simple effect but a process resulting from the gradual influence of the cognitive system.

## STRENGTHS, LIMITATIONS AND RECOMMENDATIONS FOR FUTURE STUDIES

### Strengths

This present study adds three key contributions to the literature. Firstly, it proposes a holistic model that links digital escape behaviours to memory processes. Secondly, it positions digital dementia not merely as an outcome variable but as a mechanism bridging cognitive decline and memory weakness. Thirdly, it argues that it is necessary to focus on mediating processes rather than direct effects while explaining the cognitive effects of digital technologies. As a result, this study may provide a theoretical foundation for developing models to explain the neurocognitive effects of digital behaviours.

### Limitations

Despite its substantial contributions to the literature, this study has certain limitations. Firstly, this study was a cross‐sectional design. Hence, it failed to draw firm conclusions about the direction of the relationships between digital fugue, digital dementia and digital amnesia and merely interpreted its findings within a relational framework. Secondly, the measurement tools used in the study were self‐report‐based. Accordingly, it should not be overlooked that participant responses might include perceptual biases, recall errors or social desirability inclinations. Additionally, variables such as age, gender and internet usage time were not included as control variables in the model. Lastly, the fact that the sample of this research is limited to university students restricted the generalizability of the findings to different age groups and sociocultural contexts. As a result, future studies are recommended to consider these limitations while interpreting the findings.

### Recommendations

The present study findings indicate that the effects of digital technologies on cognitive and memory processes should be considered not only in terms of duration or addiction level but also in the context of digital escapism tendencies and the erosion of cognitive functions these tendencies cause. In line with these findings, the present study recommends the following for future studies:

- Identifying and addressing digital fugue behaviour is primarily crucial in psychoeducational and preventive intervention programs designed for university students and young adults. Interventions should extend beyond simply focusing on reducing screen time and aim to comprehend the situations, purposes and emotional needs that lead individuals to turn to digital environments. Such a strategy may contribute to altering the avoidance‐based coping strategies underlying digital escape.
- Given the mediating role of digital dementia, it is necessary to prioritize interventions focused on supporting cognitive functions, specifically attention‐based, executive function‐based and mental sustainability‐based practices. Additionally, digital amnesia should be regarded as a pattern related to the transfer of cognitive responsibilities to digital settings rather than merely a memory issue. In this context, it is essential to promote learning and study techniques that enhance individuals' ability to mentally process, recall and internalize information.
- Future studies analysing the relationship among digital fugue, digital dementia and digital amnesia variables using longitudinal designs will significantly contribute to an explicit understanding of causal patterns. Additionally, studies across different age groups, occupational categories, and cultural contexts may test the generalizability of this mechanism.

## CONCLUSION

This study proposed a mechanism‐based approach to explain digital technology‐related cognitive outcomes and addressed the relationships between digital fugue, digital dementia and digital amnesia holistically. The study findings indicated that digital fugue is not merely a transient distraction or the result of excessive technology use; rather, it suggested that individual tendencies to escape from environmental, social and emotional stimuli might lead to cascading effects on cognitive and memory processes. One of the most significant outcomes of this study is that it extended beyond treating digital amnesia as a direct outcome variable and highlighted the existence of a cognitive mediating mechanism (digital dementia). In this context, digital dementia serves as a functional bridge between digital fugue and memory‐based impairments, elucidating how the erosion of cognitive functions caused by digital escape behaviours over time is reflected in memory processes. As a result, these findings reveal that it is necessary to extend beyond usage time and addiction‐focused approaches frequently emphasized in the digital behaviour‐related literature and consider behavioural patterns that indicate the nature of an individual's relationship with technology. It is recommended that these findings be utilized by researchers in the development of intervention programmes.

## AUTHOR CONTRIBUTIONS

**Deniz S. Yorulmaz‐Demir:** Conceptualization; methodology; writing – original draft; investigation; validation; software; resources; data curation; project administration; funding acquisition. **Yalçın Kanbay:** Conceptualization; methodology; software; writing – review and editing; visualization; investigation; formal analysis; resources; data curation; supervision.

## FUNDING INFORMATION

This study was supported within the scope of Artvin Çoruh University Scientific Research Projects (Project No: 2025.M83.02.05).

## CONFLICT OF INTEREST STATEMENT

The authors declare that there are no conflicts of interest to declare.

## ETHICS STATEMENT

Prior to the study, ethical approval was obtained from the Ethics Committee (Artvin Çoruh University, Decision No: E‐18457941‐050.99‐202044, Date: 20.11.2025), and institutional permission was granted by the university where the research was conducted (Artvin Çoruh University, Decision No: E‐82587833‐605‐202719, Date: 26.11.2025). During the data collection process, participants were informed about the aim and content of the study, the voluntary nature of participation and their right to withdraw from the study at any time without any consequences. No personal identifying information was requested from the participants, and the collected data were used solely for scientific purposes. The study was conducted in accordance with the principles of the Declaration of Helsinki (World Medical Association, 2013).

## PATIENT CONSENT STATEMENT

The patient consent statement is not applicable.

## Supporting information


**File S1.** Artvin Çoruh University‐ Scientific Research Projects.

## Data Availability

The data that support the findings of this study are available from the corresponding author upon reasonable request.
