# Supplementary material for: From digital escape to memory impairment: The mediating role of digital dementia in the effect of digital fugue on digital amnesia
Source: Psychol Psychother. 2026 May 15;99(3):1176–94. doi: 10.1111/papt.70077 (PMC13456396; doi:10.1111/papt.70077)
Supplement: Supplementary file 1 [file PAPT-99-1176-s001.docx]

**Supplementary File: Data Collection Tools**

**Dear Participant,**

This questionnaire has been prepared to evaluate your patterns of technological device use and the relationships among these characteristics. The form consists of a sociodemographic information section and three scales. There are no right or wrong answers to the questions; therefore, we kindly ask you to respond to them according to your own situation. Please note that your responses will affect the reliability of the study.

Thank you for your participation.

1. Gender: Female  2) Male

2. Age: ………………………………………

3. Grade/Class: ………………………………………

4. Do you have your own room? Yes  2) No

5. What do you most frequently turn to when you feel mentally or emotionally overwhelmed?

2. Digital devices
3. Talking to my family
4. Physical activity
5. Sleeping

6. Do you use digital devices such as a phone or computer to escape from environments you find boring during the day (e.g., classes, family gatherings, crowded settings)? Yes  2) No  3) Sometimes

7. On average, how many hours per day do you use a smartphone? ……………hours/day

8. Do you feel the need to check your device immediately when you receive a notification? Yes  2) No  3) Sometimes

9. Do you feel uneasy when your phone is on silent mode? 1) Yes  2) No  3) Sometimes

10. Do you keep your phone constantly on to avoid missing notifications? 1) Yes  2) No  3) Sometimes

**OPIZA Digital Fugue Scale**
Please indicate how often you use a smartphone or internet-connected devices in the following situations.

|  | **Items** | **Never (0)** | **Rarely (1)** | **Sometimes (2)** | **Often (3)** | **Always (4)** |
| --- | --- | --- | --- | --- | --- | --- |
| **1** | To avoid unexpected encounters |  |  |  |  |  |
| **2** | When I feel uncomfortable in a crowded environment |  |  |  |  |  |
| **3** | When I do not want to make eye contact with others |  |  |  |  |  |
| **4** | In environments where I feel social pressure |  |  |  |  |  |
| **5** | When I want to distance myself from people around me |  |  |  |  |  |
| **6** | When I want to forget problems in the real world |  |  |  |  |  |
| **7** | When I am unable to cope with problems |  |  |  |  |  |
| **8** | When I feel emotionally distressed |  |  |  |  |  |
| **9** | When I do not want to see what is happening around me |  |  |  |  |  |
| **10** | When I feel lonely in a social environment |  |  |  |  |  |

**Digital Dementia Scale**
Below is a list of various conditions that may arise as a result of your screen time or digital device use. Please indicate how often you experience each of these situations, selecting the option that best reflects you.

|  | **Digital Device:** Digital tools such as smartphones, computers, tablets, televisions, etc. | **Never (0)** | **Rarely (1)** | **Sometimes (2)** | **Often (3)** | **Always (4)** |
| --- | --- | --- | --- | --- | --- | --- |
| **1** | Postponing daily tasks |  |  |  |  |  |
| **2** | Spending prolonged time in front of screens |  |  |  |  |  |
| **3** | Irregular, excessive, or insufficient eating and drinking |  |  |  |  |  |
| **4** | Irregular, excessive, or insufficient sleep |  |  |  |  |  |
| **5** | Spending time in front of screens until late hours |  |  |  |  |  |
| **6** | Lower back, back, or neck pain |  |  |  |  |  |
| **7** | Headaches or eye strain |  |  |  |  |  |
| **8** | Difficulty concentrating |  |  |  |  |  |
| **9** | Mental fatigue |  |  |  |  |  |
| **10** | Physical fatigue |  |  |  |  |  |
| **11** | Decreased face-to-face social interaction |  |  |  |  |  |
| **12** | Feeling more restless or anxious |  |  |  |  |  |
| **13** | Feeling more depressed |  |  |  |  |  |
| **14** | Difficulty remembering daily information or tasks |  |  |  |  |  |
| **15** | Difficulty learning or recalling new information |  |  |  |  |  |

**Digital Amnesia Scale**
Please read the following statements and mark the option that best reflects you.

|  | **Digital Device:** Digital tools such as smartphones, computers, tablets, televisions, etc. | **Never (0)** | **Rarely (1)** | **Sometimes (2)** | **Often (3)** | **Always (4)** |
| --- | --- | --- | --- | --- | --- | --- |
| **1** | I use digital reminders for my scheduled tasks. |  |  |  |  |  |
| **2** | I have difficulty remembering daily tasks without my digital devices. |  |  |  |  |  |
| **3** | I take notes on my digital devices when learning new information. |  |  |  |  |  |
| **4** | I have difficulty remembering information without my digital devices. |  |  |  |  |  |
| **5** | I prefer to quickly access information using my digital device rather than remembering it. |  |  |  |  |  |
| **6** | Knowing where I have stored information is more important to me than remembering it. |  |  |  |  |  |
| **7** | Accessing information quickly through my digital devices is more convenient for me. |  |  |  |  |  |
| **8** | I find it easier to store information on digital devices than to remember it. |  |  |  |  |  |
| **9** | I prefer taking digital notes rather than physical notes to remember information. |  |  |  |  |  |
| **10** | I use the internet to access information instead of keeping it in my memory. |  |  |  |  |  |
| **11** | Thanks to my digital devices, I do not worry about forgetting information. |  |  |  |  |  |
| **12** | I rely more on digital devices than on my own memory to retain information. |  |  |  |  |  |
